# Overlapping community detection in weighted networks via hierarchical clustering

**DOI:** 10.1371/journal.pone.0312596

**Published:** 2024-10-28

**Authors:** Petr Prokop, Pavla Dráždilová, Jan Platoš

**Affiliations:** Department of Computer Science, FEECS, VŠB - Technical University of Ostrava, Ostrava, Czech Republic; University of Porto Faculty of Engineering: Universidade do Porto Faculdade de Engenharia, PORTUGAL

## Abstract

In real-world networks, community structures often appear as tightly connected clusters of nodes, with recent studies suggesting a hierarchical organization where larger groups subdivide into smaller ones across different levels. This hierarchical structure is particularly complex in trade networks, where actors typically belong to multiple communities due to diverse business relationships and contracts. To address this complexity, we present a novel algorithm for detecting hierarchical structures of overlapping communities in weighted networks, focusing on the interdependency between internal and external quality metrics for evaluating the detected communities. The proposed Graph Hierarchical Agglomerative Clustering (GHAC) approach utilizes maximal cliques as the basis units for hierarchical clustering. The algorithm measures dissimilarities between clusters using the minimal closed trail distance (*CT*−distance) and the size of maximal cliques within overlaps, capturing the density and connectivity of nodes. Through extensive experiments on synthetic networks with known ground truth, we demonstrate that the adjusted Silhouette index is the most reliable internal metric for determining the optimal cut in the dendrogram. Experimental results indicate that the GHAC method is competitive with widely used community detection techniques, particularly in networks with highly overlapping communities. The method effectively reveals the hierarchical structure of communities in weighted networks, as demonstrated by its application to the OECD weighted trade network, which describes the balanced trade value of bilateral trade relations.

## 1 Introduction

Detecting hierarchical overlapping communities in undirected and weighted complex networks is crucial for understanding the diverse structures in real-world networks, where nodes often participate in multiple communities organized in a nested, hierarchical manner. For example, in biological networks, proteins may function in multiple pathways or complexes, with these pathways further subdividing into smaller functional units. In economic trade networks, countries form overlapping trade blocks organized into larger economic zones, with subgroups defined by specific trade agreements or regional proximity.

Communities in complex networks are subsets of vertices within which vertex–vertex connections are dense, but between which connections are less dense [1]. This structural property, known as community structure, reflects the inhomogeneous distribution of edges both globally and locally, with high concentrations of edges within special groups of vertices, and low concentrations between these groups [2]. Traditional approaches often focus on identifying non-overlapping communities, where each node belongs to a single group, optimizing criteria such as modularity to detect these tightly-knit regions within the network.

However, real-world networks frequently exhibit overlapping community structures, where nodes participate in multiple communities simultaneously. In this context, Palla et al. [3] introduced the concept of *k*-clique communities, where a community is defined as a union of all *k*-cliques (fully connected subgraphs of size *k*) that are interconnected through adjacent *k*-cliques sharing *k* − 1 nodes. This definition highlights the interweaving nature of communities, which often overlap and share significant portions of their nodes.

Yang and Leskovec [4] further examined the nature of overlapping communities, revealing that these overlaps tend to be more densely connected than the non-overlapping parts of the network. This finding challenges the traditional view that community overlaps are less cohesive, suggesting that overlapping regions may serve as integrative hubs, facilitating interactions across different functional groups.

Nested communities in complex networks are essential for understanding their multi-level structure. Hierarchical detection uncovers both small sub-communities and larger groups, allowing analysis at different scales [5]. The significance of identifying nested communities is underscored by findings in biological networks, where cross-scale structural relationships demonstrate considerable nestedness in both empirical and simulated contexts [6].

Despite the abundance of community detection algorithms, few methods effectively integrate the identification of overlapping communities and their hierarchical structure, particularly in the context of weighted networks. This study addresses this gap by proposing a novel approach that combines these two perspectives. Additionally, we extend our methodology with evaluation using synthetic networks from the LFR benchmark, focusing on the relationship between internal and external community quality metrics.

Our point of view on the concept of uncovering community structure is based on the recognition that cliques are the densest subgraphs and are always part of a community. By aggregating groups of cliques close from a distance perspective into a single community, we effectively define a community as a group of closely connected cliques. This approach leverages the structural properties of cliques to capture the dense and overlapping nature of communities within biconnected parts of networks.

The proposed algorithm is designed to identify hierarchical overlapping communities in undirected and weighted complex networks. This study finds community by merging elements based on the minimal closed trail distance (*CT*-distance) [7]. The fundamental units for clustering are cliques that may overlap. The *CT*-distance metric quantifies the dissimilarity between nodes, determining the separation of communities in the algorithm. The overlap in the proposed algorithm is provided by cliques that are base elements for hierarchical clustering. Using cliques as the basis elements that enter the clustering process ensures that the nodes in a clique will always be in a common community.

This paper’s main contributions can be summarized as:

- Robust methodology for overlapping hierarchical community detection in undirected weighted networks.
- Proposition of the adjusted Silhouette index for the optimal network cover selection process based on *CT*−distance.
- Comprehensive analysis of internal quality metrics for community evaluation, identifying the Silhouette index as highly correlated with external evaluations.
- Validation of the GHAC method as a competitive algorithm independent of neural networks and modularity optimization techniques.
- Case study demonstrating the identification of nested community structures by a dendrogram, with local optima in multiple quality criteria (Silhouette index, modularity, and conductance) uncovering the nestedness of communities in the trade network.

This paper is organized as follows. Section Related work concentrates on overlapping and hierarchical community detection. Next, the proposed algorithm for detecting overlapping hierarchical communities in the weighted network is described. Section Experiments contains a brief review of the selection criteria for identifying the best communities, experiments on synthetic networks (LFR), and a Case study: trade network of OECD countries. The Conclusion summarises new findings on detecting overlapping communities in a weighted network.

## 2 Related work

Community detection in complex networks has been a topic of significant research over the past decades. Early studies focused on detecting non-overlapping communities, where each node belongs to a single group. Methods like the Girvan-Newman algorithm [1] and modularity optimization approaches [8] laid the foundation for detecting clear-cut divisions in networks. However, real-world networks often exhibit more intricate structures where nodes belong to multiple communities, motivating the development of algorithms for overlapping community detection. Our method builds upon two key areas of research, hierarchical community detection and overlapping community detection. The related work first explores these two domains separately, followed by a review of methods that integrate both approaches at the same time, as our introduced method combines hierarchical and overlapping community detection.

Most real-world networks exhibit overlapping and nested community structures, where nodes belong to multiple communities, and these communities are organized hierarchically [3]. Such modular and hierarchical structures are pervasive in complex networks, reflecting the multifaceted nature of interactions within these systems. For instance, large clusters composed of smaller, more cohesive sub-clusters often signal a hierarchical organization within a network, a characteristic feature of many complex systems [9]. Hierarchical community structures extend beyond simple clustering, incorporating organization across multiple scales within a network.

Hierarchical agglomerative methods for community detection employ various strategies to calculate dissimilarity or similarity between communities. For instance, Castrillo et al. [10] use modified structural similarity (cosine similarity) in their approach, Berahmand et al. [11] apply extended Jaccard similarity, and Brzozowski et al. [12] utilize the Wasserman-Faust distance to detect communities effectively.

In addition to these traditional methods, statistical approaches like the stochastic block model (SBM) [13–15] provide a probabilistic framework for community detection that is particularly suited for hierarchical structures. Stochastic block models enable the modeling of networks with probabilistic community assignments, allowing for both hard and soft partitioning of nodes into communities. Amini et al. [16] extend this concept by using a hierarchical stochastic block model for community detection in multiplex networks. Schaub et al. [17] further develop this approach by introducing a hierarchical definition based on stochastic externally equitable partitions, which helps in detecting hierarchical structures efficiently through the examination of spectral properties.

In parallel, overlapping community detection focuses on identifying communities where nodes can belong to multiple groups, reflecting the reality of many complex networks. Clique-based methods, such as the Clique Percolation Method (CPM) introduced by Palla et al. [3], are particularly effective for this task. CPM defines a community as a union of all k-cliques that can be reached through a series of adjacent k-cliques, with extensions like those by Farkas et al. [18] adapting the method for weighted networks. Another significant contribution to this area is the Clique-Based Louvain Algorithm (CBLA), which enhances CPM by addressing the issue of unclassified nodes that are not part of any clique, integrating them into communities using the Louvain method [19]. Maximal cliques are used for the set-covering approach to overlapping community detection in [20]. Other methods for overlapping community detection, such as density peak clustering (DPClus) [21] and its variants like IPCA [22], identify communities based on subgraph density and connectivity properties.

Non-negative matrix factorization (NMF) also plays a key role in community detection. For instance, Ye et al. [23] introduced the Deep Autoencoder-like NMF (DANMF), which leverages the feature representation capabilities of deep autoencoders for overlapping community detection in weighted networks. Another method, WSNMF, utilizes weighted symmetric NMF to detect communities in attributed graphs [24]. The effectiveness of NMF-based approaches can be evaluated using reconstruction error, clustering metrics like the Dunn index [25], and another embedding-based measure like Silhouette score [26], which assesses the quality of clustering.

The integration of hierarchical and overlapping community detection is essential for capturing the full complexity of real-world networks. For example, Lancichinetti et al.’s Order Statistics Local Optimization Method (OSLOM) [27] represents a comprehensive approach, capable of detecting communities while considering edge directions, weights, overlaps, hierarchies, and dynamics. Similarly, stochastic block models (SBM) provide a powerful statistical framework for community detection, enabling the analysis of clustering dynamics and community structures in networks with overlapping and hierarchical features [28, 29].

Overall, the accurate detection and evaluation of community structures, whether hierarchical, overlapping, or both, are crucial for understanding the underlying patterns in complex networks. A variety of metrics, including Overlapping Normalized Mutual Information (ONMI), F1-scores, Omega index, likelihood-based criteria, and embedding-based measures, provide a comprehensive toolkit for assessing the performance of community detection methods. Comparative studies and evaluations, such as those discussed in [30–32], offer valuable insights into the strengths and limitations of different approaches across diverse networks and community structures, highlighting the importance of community detection in complex networks.

## 3 Preliminaries

This section briefly introduces the necessary preliminary knowledge for this work, which includes the basic notation, a problem statement for community detection, and a formal definition of *CT*−distance used throughout this paper.

We define a weighted undirected graph as *G* = (*V*, *E*, *w*), where *V* = {*v*_1_, …, *v*_*n*_} represents the set of nodes, with *n* denoting the total number of nodes. The set of edges is *E*, where *e*_*ij*_ = (*v*_*i*_, *v*_*j*_)∈*E* represents an edge between nodes *v*_*i*_ and *v*_*j*_, and the total number of edges is |*E*| = *m*. The function $w:E\to R^{+}$ assigns positive integer weights to the edges. To capture the graph’s structure, we utilize the adjacency matrix *A* = (*a*_*ij*_)_*n*×*n*_, which reflects the presence or absence of edges, and the weighted adjacency matrix *W* = (*w*_*ij*_)_*n*×*n*_, which encodes the weights of the edges between nodes.

### 3.1 *CT*−distance in graph

The *CT*-distance is used to quantify the structural closeness of nodes in a graph based on a closed trail.

**Definition 3.1** *Let G* = (*V*, *E*) *be a graph. Let* $d_{CT}:V\times V\to R_{0}^{+}$ *be defined by the equation*

$$d_{CT}\left(u,v\right)=min_{CT(u,v)\in G}\left|CT\left(u,v\right)\right|,$$

*where CT*(*u*, *v*) *is a closed trail that contains the vertices u*, *v*. *Then the function d*_*CT*_ *is called the closed trail distance* (*CT-distance*).

The definition of the *CT*−distance may be extended for the weighted graph *G* = (*V*, *E*, *w*). The weights of edges are considered like the similarity in the sense that a greater value is better. We need the reciprocal value of *w*(*e*) to express the dissimilarity between vertices such that the weight of the closed trail will be determined more by weights than by length.

**Definition 3.2** *Let G* = (*V*, *E*, *w*) *be a weighted graph with w*(*e*) ∈ 〈1, *s*〉 *for all e* ∈ *E*, *s* ≥ 1 *and let the mapping* $d_{wCT}:V\times V\to R_{0}^{+}$ *be defined by the equation*

$$d_{wCT}\left(u,v\right)=min_{CT(u,v)\subseteq G}(\sum_{\forall e\in CT(u,v)}\frac{1}{w(e)}).$$

*Then the function d*_*wCT*_ *is called the weighted closed trail distance* (*wCT*−*distance*).

The *CT*−distance between nodes *u* and *v* corresponds to the length of the shortest closed trail containing nodes *u* and *v* in the undirected graph. This distance measure considers the graph’s structure and reflects the neighborhood density around the nodes. The closed trail distance for weighted networks (*wCT*−distance) prefers a closed trail with the smallest weight, achievable by a shorter length or smaller edge weights in the trail. The original edge weight represents the similarity between vertices and is converted to dissimilarity for weighted *CT*−distance calculation.

### 3.2 Concept of community

A common approach to community definition is based on edge density. A community is characterized as a subgraph with an edge density greater than the edge density between communities.

In this paper, our concept of the community is a subgraph with the smallest possible *CT*−distance between community nodes. The distance between communities is greater than or at most equal to the distance between nodes in the community.

Our approach leverages maximal cliques, which are the densest substructures and equivalently substructures with the smallest nonzero *CT*−distance between vertices in a network, as the fundamental units of community formation that can not be divided only overlapped. Cliques represent fully connected groups of nodes, and by merging closely connected cliques based on their *CT*−distance, we identify overlapping communities that reflect both tight-knit local relationships and broader global interactions.

## 4 Proposed algorithm for overlapping hierarchical community detection in weighted networks

Hierarchical agglomerative clustering on the graph (GHAC) detects nested communities in a network using novel dissimilarity. The edge weight is incorporated into the calculation of *wCT*-distance [7] that, together with an overlap of cliques, determines dissimilarity between clusters during the GHAC. Maximal cliques are the base elements of the GHAC. This core idea has been presented in [33], including the conceptual details and some analogy to CPM. The main contribution of this paper is the extension of the community detection method to weighted networks and improving the methodology of selecting the best cut in the dendrogram.

**Fig 1:**
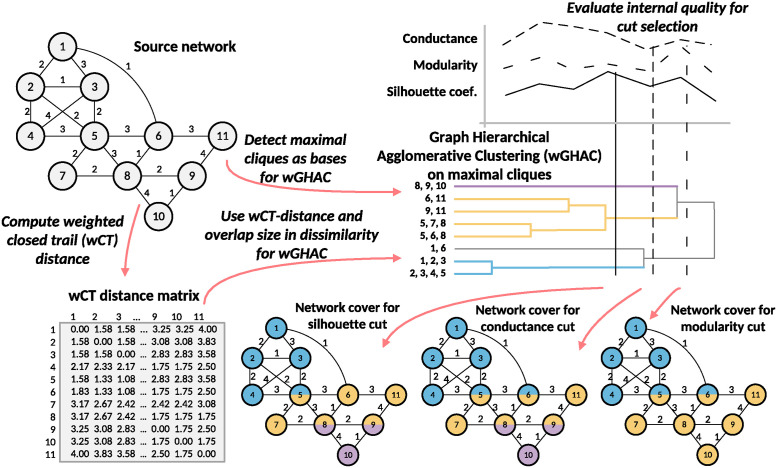
Overview of the proposed methodology for community detection in weighted networks.

Fig 1 depicts the sequence of operations in our community detection methodology tailored for weighted networks. Initially, we compute the weighted closed trail (wCT) distance, followed by the detection of maximal cliques to serve as the basis for our hierarchical agglomerative clustering. We evaluate various cuts within the generated hierarchy by monitoring internal quality metrics to produce the most coherent network cover.

### 4.1 Dissimilarities for graph hierarchical agglomerative clustering

We introduce dissimilarities between clusters based on the *wCT*−distance, and the weight of overlap in a weighted graph. We consider that the weight of overlap will be represented by the densest part of overlap, which is a clique. The weight of the densest clique in the overlap of two communities can be formalized by the biggest and the heaviest clique as:

$$\bar{w}\left(Q\right)=\left|Q\right|+\frac{\sum_{\forall e\in Q}w\left(e\right)}{\sum_{\forall e\in E}w\left(e\right)}.$$

We define dissimilarities based on the Complete Linkage (CL) and the Average Linkage (AL) approach for GHAC on the weighted graph *G* = (*V*, *E*, *w*) as:

$$d_{wGHAC}^{CL}\left(C_{i},C_{j}\right)=\frac{max_{\left(v_{i}\in C_{i}\backslash C_{j}\right),\left(v_{j}\in C_{j}\backslash C_{i}\right)}d_{wCT}\left(v_{i},v_{j}\right)}{1+argmax_{Q\in C_{i}\cap C_{j}}\bar{w}\left(Q\right)},$$

and

$$d_{wGHAC}^{AL}\left(C_{i},C_{j}\right)=\frac{\sum_{\left(v_{i}\in C_{i}\backslash C_{j}\right),\left(v_{j}\in C_{j}\backslash C_{i}\right)}d_{wCT}\left(v_{i},v_{j}\right)}{|\left(C_{i}\cup C_{j}\right)\backslash \left(C_{j}\cap C_{i}\right)|(1+argmax_{Q\in C_{i}\cap C_{j}}\bar{w}\left(Q\right))}.$$

For the current work, we have denoted the use of the GHAC method with dissimilarity $d_{wGHAC}^{AL}$ as wAL GHAC (Average linkage hierarchical clustering on the weighted graph) and for dissimilarity $d_{wGHAC}^{CL}$ as wCL GHAC.

### 4.2 Community detection procedure

A brief description of the steps in the proposed community detection method is given in Algorithm 1. Suurballe’s algorithm [34] is used to calculate *wCT*−distances among vertices. These distances represent one part of the dissimilarity utilized in the GHAC. The overlap size represents the other part of dissimilarity, as explained in the previous section.

The internal quality criteria used in Step 3.4. are described in Section 4.3.

**Algorithm 1:** Proposed community detection method based on the GHAC and dissimilarity leveraging *wCT*−distance.

**Input**: The 2-edge-connected component of a network (i.e., a graph without bridges)

**Output**: Network cover

**Step 1:** Calculate *wCT*−distance matrix among vertices in a input graph.

**Step 2:** Find maximal cliques (Bron-Kerbosch alg. [35]).

**Step 3:** Hierarchical agglomerative clustering on the graph:

 **Step 3.1:** Agglomerate communities according to proposed dissimilarity with maximal cliques as base elements.

 **Step 3.2:** Map merged clusters of base elements to origin graph vertices.

 **Step 3.3:** Filter out small clusters and fill in the network cover.

 **Step 3.4:** Evaluate network cover structural quality by internal criteria for quality evaluation.

 **Step 3.5:** Repeat the algorithm from Step 3.1 until all clusters are merged.

**Step 4:** Choose the best level for a cut of a dendrogram of agglomerative steps.

Maximal cliques are used as bases in the GHAC, and merged clusters of maximal cliques are mapped to vertices using a few post-processing steps. The communities with a size less than 5 are removed from network cover during the post-processing. Any vertices that do not belong to any community are assigned to one of the most frequent communities among its neighbors. The repository with the implementation of the proposed method is available on GitHub, which can be accessed at the following link: https://github.com/petr-prokop/weighted_graph_hierarchical_agglomerative_clustering.

### 4.3 Internal quality criteria for the optimal cut selection

The effectiveness of the GHAC methodology, as delineated in Algorithm 1, depends on selecting an optimal dendrogram cut to ensure a high-quality network cover. Contrary to preliminary observations in [36], where modularity *M*^*ov*^ failed to reliably signal the optimal dendrogram cut, alternative internal evaluation metrics are considered for choosing better network cover. The internal quality evaluation criteria refer to methods that assess community structure based on information derived from the network itself, such as modularity and conductance, or through dissimilarity measures calculated on the graph. In contrast, external validation criteria rely on additional information about the community structure, such as ground truth. Internal criteria are applicable to real-world data, while external criteria are primarily used for comparison on synthetic benchmarks.

The study by Chakraborty et al. [37] provides a comprehensive review of internal metrics correlating with external validation metrics for overlapping community structures. We have adopted two divergent modularity definitions [38, 39] tailored for overlapping communities within the GHAC’s agglomerative framework, with roots in Newman’s foundational modularity approach [40].

- Shen’s modularity [38] adapts to the overlapping community paradigm by accounting for vertex memberships in multiple communities. It seamlessly reverts to Newman’s original modularity for singular community membership per vertex, as articulated:
  $$M^{e}=\frac{1}{2m}\sum_{k=1}^{c}\sum_{i,j\in C_{k}}\frac{1}{O_{i}O_{j}}[w_{ij}-\frac{wdeg(i)wdeg(j)}{2m}],$$
  where *O*_*v*_ denotes the community count for vertex *v*, and *c* represents the total community number.
- Lazar’s modularity metric [39] proposes an alternative measure, underpinning two core assumptions: nodes predominantly share edges within their community, and communities themselves should manifest as densely interconnected. This metric is expressed as:
  Mov=1c∑k=1c(∑i∈Ck∑j∈Ck,i≠jwij-∑j∉Ckwijwdeg(i)OinCke|Ck|(|Ck|2)),
  with *c* indicating the cluster count, *O*_*i*_ the cluster membership count for node *i*, |*C*_*k*_| the node count, and $n_{C_{k}}^{e}$ the edge count within the *k*th cluster *C*_*k*_.

Metrics from [41, 42] are reformulated for weighted graphs *G* = (*V*, *E*, *w*), where *C*_*i*_ ⊂ *V* induces community, *n* = |*V*|, *n*_*i*_ = |*C*_*i*_|, *m*_*i*_ = |{(*v*_*i*_, *v*_*j*_)∈*E*;*v*_*i*_, *v*_*j*_ ∈ *C*_*i*_}|, *N*_*i*_ = {*v*_*j*_ ∈ *V*;(*v*_*i*_, *v*_*j*_)∈*E*}, $wcut\left(C_{i}\right)=\sum_{v_{i}\in C_{i}}\sum_{v_{j}\notin C_{i}}w_{ij}$, and $wvol\left(C_{i}\right)=\sum_{v_{i}\in C_{i}}\sum_{v_{j}\in V}w_{ij}$. We applied selected metrics—conductance, expansion, internal (edge) density, ratio cut, normalized cut, and Flake ODF—on communities *C*_*i*_:

- Conductance: $Cond_{w}\left(C_{i}\right)=\frac{wcut(C_{i})}{wvol(C_{i})}$,
- Expansion: $Ex_{w}\left(C_{i}\right)=\frac{wcut(C_{i})}{n_{i}}$,
- Internal density: $InD_{w}\left(C_{i}\right)=\frac{wvol\left(C_{i}\right)-wcut\left(C_{i}\right)}{n_{i}\cdot \left(n_{i}-1\right)}$,
- Ratio cut: $RC_{w}\left(C_{i}\right)=\frac{wcut(C_{i})}{n_{i}\cdot \left(n-n_{i}\right)}$,
- Normalized cut: $NC_{w}\left(C_{i}\right)=\frac{wcut(C_{i})}{wvol(C_{i})}+\frac{wcut(C_{i})}{wvol(V\backslash C_{i})}$,
- Flake ODF: $FODF_{w}\left(C_{i}\right)=\frac{\left|\{i\in C_{i};\sum_{j\in C_{i}}w_{ij}\leq \frac{1}{2}\sum_{j\in V}w_{ij}\}\right|}{n_{i}}$.

One metric from [43] for unweighted graphs is used as well:

- Internal transitivity: $InT\left(C_{i}\right)=\frac{1}{n_{i}}\sum_{i\in C_{i}}\frac{\sum_{j,k\in N_{i}}a_{jk}}{k_{int}\left(i\right)\cdot \left(k_{int}\left(i\right)-1\right)}$, where *k*_*int*_(*i*) = |*N*_*i*_∩*C*_*i*_|.

Standard methods for evaluation of clustering quality are reformulated for application on the weighted graph *G* = (*V*, *E*, *w*) and overlapping clustering as the Dunn index and Silhouette index.

- Dunn index
  $$DI\left(C\right)=\frac{min_{C_{i},C_{j}\in C}d_{wGHAC}^{XL}\left(C_{i},C_{j}\right)}{max_{C_{i}\in C}diam\left(C_{i}\right)},$$
  where $C=\{C_{1},\ldots ,C_{k}\}$ is the set of communities covered vertices, $d_{wGHAC}^{XL}\left(C_{i},C_{j}\right)$ represent dissimilarity (SL, CL or AL) between communities, and diameter of community is $diam\left(C_{k}\right)=max_{u,v\in C_{k}}d_{wCT}\left(u,v\right)$.
- Silhouette index
  $$SI\left(C\right)=\frac{1}{\left|V\right|}\sum_{i=1}^{\left|V\right|}s_{i},$$
  $$s_{i}=\frac{1}{\left|P\right|}\sum_{p\in P}\frac{b_{i}-a_{i}^{p}}{max\{a_{i}^{p},b_{i}\}},$$
  $$b_{i}=min_{k;u_{i}\notin C_{k}}\frac{1}{|C_{k}|}\sum_{u_{k}\in C_{k}}d_{wCT}\left(u_{i},u_{k}\right),$$
  $$a_{i}^{p}=\frac{1}{|C_{p}|-1}\sum_{u_{j}\in C_{p}}d_{wCT}\left(u_{i},u_{j}\right),$$
  $$P=\{p;\;u_{i}\in C_{p}\}.$$

### 4.4 Proposed method demonstration

In article [44], the authors identified communities in a small network as *C* = {{0, 1, 2, 3, 4, 5}, {6, 7, 8, 9}, {5, 6, 10}, {10, 11, 12}} with corresponding internal quality metrics of *M*^*e*^ = 0.24, *M*^*ov*^ = 0.40, and *SI* = 0.38.

**Fig 2:**
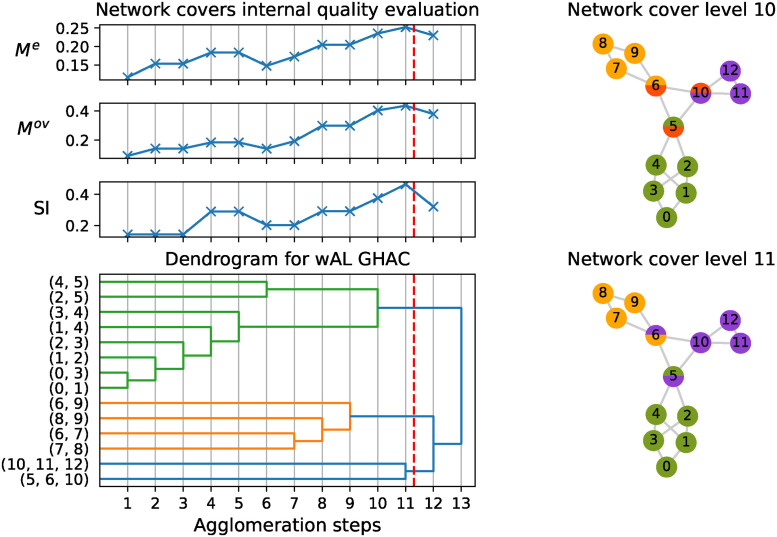
Illustration of the wAL GHAC algorithm on a small network. The dendrogram shows the hierarchical merging of cliques into communities. Internal evaluation metrics (*M*^*e*^, *M*^*ov*^, *SI*) guide the selection of the optimal dendrogram cut (red and purple dashed lines) for the best network cover. Overlapping communities for steps 10 and 11 are illustrated.

Fig 2 presents the results of applying the wAL GHAC algorithm to the same network, with a post-processing adjustment enforcing a minimum community size of three nodes and disabling label propagation for clarity. The dendrogram illustrates the hierarchical agglomeration of clusters.

While the communities identified in [44] are represented within the wAL GHAC hierarchy, our method achieves superior internal quality metrics at different levels. Specifically, at level 11, *M*^*e*^ = 0.25, *M*^*ov*^ = 0.43, and *SI* = 0.46 indicate an optimal network cover, surpassing previous results and providing a more refined structure.

## 5 Experiments

This study aims to evaluate the efficacy of both novel and conventional algorithms in detecting communities characterized by significant overlaps and multiple community memberships. The parameters for the LFR benchmark [45] were carefully chosen to replicate real-world complexities, such as overlapping communities and varied node degrees, thereby providing a realistic setting for algorithm assessment.

Building upon previous findings [36], this research assesses the performance of the community detection method by employing an unweighted version of the LFR benchmark for initial graph generation, followed by a controlled process of assigning weights sourced from a normal distribution and subsequently adjusted to emphasize intra-community edges. Despite the availability of LFR benchmark versions that inherently support weighted graph generation, this study uses artificial weight assignment to investigate irregularities observed in the weight distribution between intra- and inter-community edges across different open-source implementations of weighted LFR versions. This approach examines algorithm performance in scenarios where intra-community connections are intentionally strengthened.

Utilizing the same synthetic benchmark networks as in [36], the analysis extends the evaluation of community partition quality, aligning with established ground truth structures and refining the identification of optimal cut in the dendrogram. This methodical examination enhances the reliability of community detection assessments and contributes to a deeper understanding of the proposed methods.

### 5.1 Experimental setup

For the performance evaluation of community detection algorithms, we generated a comprehensive set of synthetic networks using the LFR benchmark. The configuration parameters for these graphs included 500 nodes, a power-law exponent for the degree distribution of −2, and a power-law exponent for the community size distribution of −1. The average and maximum degrees were varied as (〈*k*〉, *k*_*max*_)∈{(10, 30), (20, 30), (10, 50), (20, 50), (30, 50)}, while the minimum and maximum community sizes were set as (*c*_*min*_, *c*_*max*_)∈{(7, 30), (15, 50)}. The number of nodes involved in overlaps was tested at *on* ∈ {0, 50, 100, 200}, with overlapping nodes holding memberships *om* ∈ {2, 4, 6}, and mixing parameters tested at *μ* ∈ {0.1, 0.2, 0.3}. Five unique graph instances were created using distinct seeds for each parameter combination.

Two subsets of these networks were utilized for experimental clarity and focus. Subset A consists of 540 selected graphs with a mixing parameter *μ* = 0.1, to assess behavior under the case of a well-defined community structure. Subset B includes 945 networks characterized by “reasonable” overlap conditions, excluding networks without overlap and networks with 40% of overlapping nodes while node’s membership *om* ≥ 4.

The quality of the detected communities was validated against the known (ground truth) community structure of the synthetic networks. The standard set of evaluation metrics consists of *ONMI*_*LFK*_ [46], *ONMI*_*MGH*_ [47], F1, NF1 [48, 49], and Omega index [50]. Additionally, the accuracy of the algorithms in identifying overlapping nodes was assessed using the Overlapping Nodes F1 score (ONF1) [51], which acts as a binary classifier to detect overlapping nodes. This rigorous evaluation framework ensures a comprehensive analysis of algorithm performance across varying degrees of community overlap and interconnectivity, providing significant insights into their performance under diverse network conditions.

### 5.2 Interrelation among external evaluation metrics for GHAC methods

This analysis explores the relation between various external evaluation metrics applied to evaluate network cover quality in GHAC methods. The analysis was performed only on the dendrogram sections where the number of detected communities was between half and twice the number of ground truth communities present in the network.

**Fig 3:**
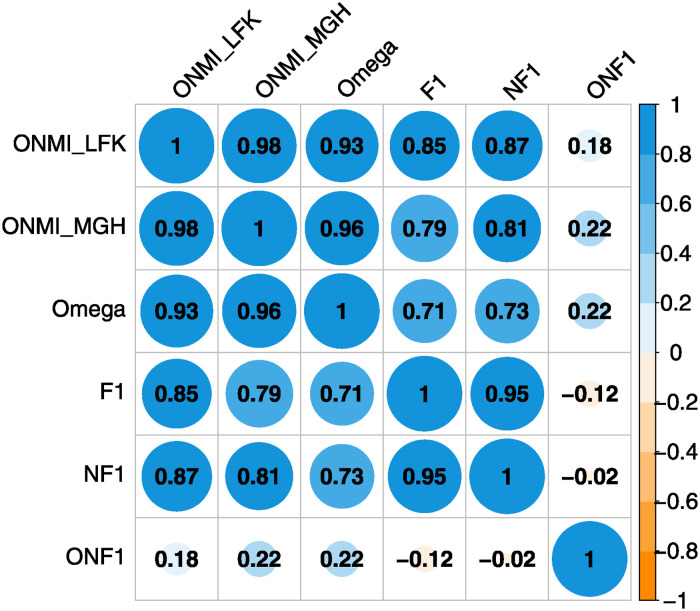
Spearman’s correlation coefficient for comparison of the external quality evaluation of community covers.

Fig 3 presents Spearman’s correlation coefficient matrix for various external quality evaluations. The *ONMI*_*LFK*_ and *ONMI*_*MGH*_ exhibit a very high correlation (*r* = 0.98), indicating similar evaluation outcomes. These metrics correlate strongly with the Omega index, suggesting they reliably reflect community structure correspondence with ground truth. The F1 and NF1 scores, while showing an expected high correlation with each other (*r* = 0.95), have a strong correlation with ONMIs and Omega indices, pointing to consistent yet distinct evaluations. ONF1 score shows a weak correlation with the other metrics, highlighting its unique contribution to understanding algorithm performance on overlapping community detection, independent of other community detection quality measures.

Given these findings, we have selected *ONMI*_*LFK*_, *NF*1, and *ONF*1 as the primary metrics for comparative analysis of absolute values across various community detection methodologies.

### 5.3 Efficiency of internal metrics in cut selection

This study aims to identify the most effective internal quality metrics for determining the optimal dendrogram partitioning in community detection. We examined the correlation between internal and external criteria. We evaluated the absolute performance metrics for various levels of GHAC methods selected by internal quality measures on a benchmark set of networks. Section 4.3 defines the set of used internal metrics. In the case of the definition of internal criteria for a single community from the network cover, the aggregation is done by averaging the values of every community from the network cover.

**Fig 4:**
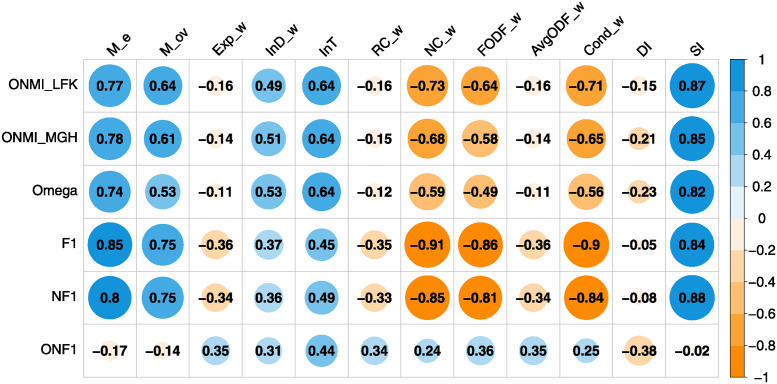
Relation of external and internal quality criteria by Spearman’s correlation coefficient.

We investigated the relationship between internal and external quality criteria for community detection on generated network sets and various hierarchical cuts of wAL GHAC and wCL GHAC methods. Fig 4 reveals a strong correlation between most external evaluations and the Silhouette index (SI). Metrics such as *ONMI*_*LFK*_, *ONMI*_*MGH*_, and Omega demonstrate a strong positive correlation with SI, modularity measures, and internal transitivity while exhibiting a strong negative correlation with the normalized cut, FODF, and conductance. Notably, the F1 and NF1 evaluations correlate highly with the normalized cut and conductance. Internal measures show a moderate correlation with the ONF1 criterion, where internal transitivity (InT) registers the highest correlation.

**Fig 5:**
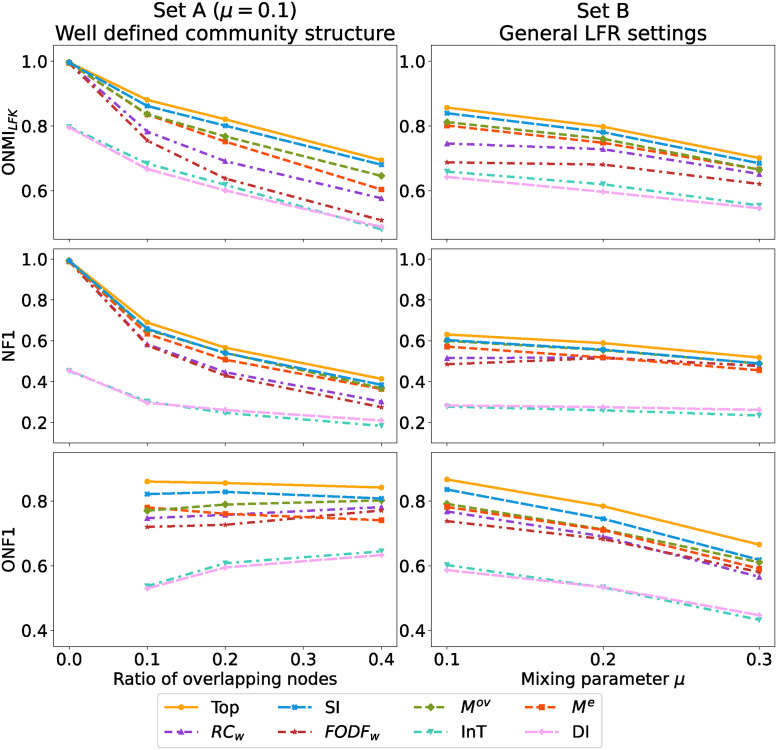
Evaluation of different internal criteria effect on the structural quality of detected network cover in wAL GHAC method. The line denoted as Top indicates the best value of external quality achieved in wAL GHAC, and the additional lines represent other internal quality measures used in the Algorithm 1.

Fig 5 compares internal quality measures using two validation sets. In set A, an observed decline in *ONMI*_*LFK*_ and NF1 scores with an increased ratio of overlapping nodes. On the contrary, ONF1 scores remain relatively stable, indicating the method’s robustness in detecting overlapping nodes even in networks with highly overlapping community structures. Validation set B suggests a slight decrease in community detection quality, as indicated by *ONMI*_*LFK*_ and NF1 scores, with higher levels of community mixing (parameter *μ*).

Variation in selection method efficacy for wAL GHAC is evident against the top evaluation value present in the hierarchy. The Silhouette index (SI) notably demonstrates the minimal disparity in *ONMI*_*LFK*_, NF1, and ONF1. Internal measures such as SI, *M*^*e*^, and *M*^*ov*^ consistently yield high scores, predicting quality network covers. In contrast, Internal Transitivity (InT) and the Dunn Index (DI) show less consistent performance, potentially leading to lower-quality community detection outcomes. Similar patterns are observed for wCL GHAC.

The experimental results demonstrate an enhancement in addressing the limitations previously identified in [36], narrowing the gap between actual and top external evaluation scores when employing SI over modularity *M*^*ov*^ for dendrogram cut selection. The Silhouette index is used for subsequent experimental comparisons with state-of-the-art methods due to its effectiveness in enhancing GHAC community detection methodologies.

### 5.4 Comparative analysis

In this comparative analysis, we explored the effectiveness of various state-of-the-art methods for community detection within weighted networks. The Silhouette index (SI) emerged as the optimal monitoring criterion for evaluating proposed GHAC methodologies as discussed in Section 5.3. The quality of detected communities was compared with a selection of algorithms. We employed standard algorithms capable of identifying overlapping communities, such as OSLOM [27], IPCA [22], and ASLPAW [52].

The analysis included the Weighted Stochastic Block Model (WSBM) implemented in graph-tool [13], which accommodates various weight’s value modeling approaches and offers variations of SBM implementations with and without degree corrections and the ability to model blocks with overlaps. In the results section, we reported only the top-performing configurations under the designation WSBM. Specifically, we denoted the performance of the degree-corrected and overlapping version with exponential or normal weight modeling as DCO-WSBM.

Additionally, our analysis included the DANMF algorithm [23], which involves multiple input parameters such as the number of detected communities—a crucial aspect often undetermined in real-world networks. A threshold value for the membership matrix [53] was introduced as an additional parameter to allow detection of overlaps by the DANMF method. A hyperparameter search was conducted, optimizing the performance in 700 iterations to find the most effective configurations. Although such detailed optimization is impractical for real-world applications, it provided clear insights into the DANMF optimal performance within the controlled environment of the LFR benchmark.

**Fig 6:**
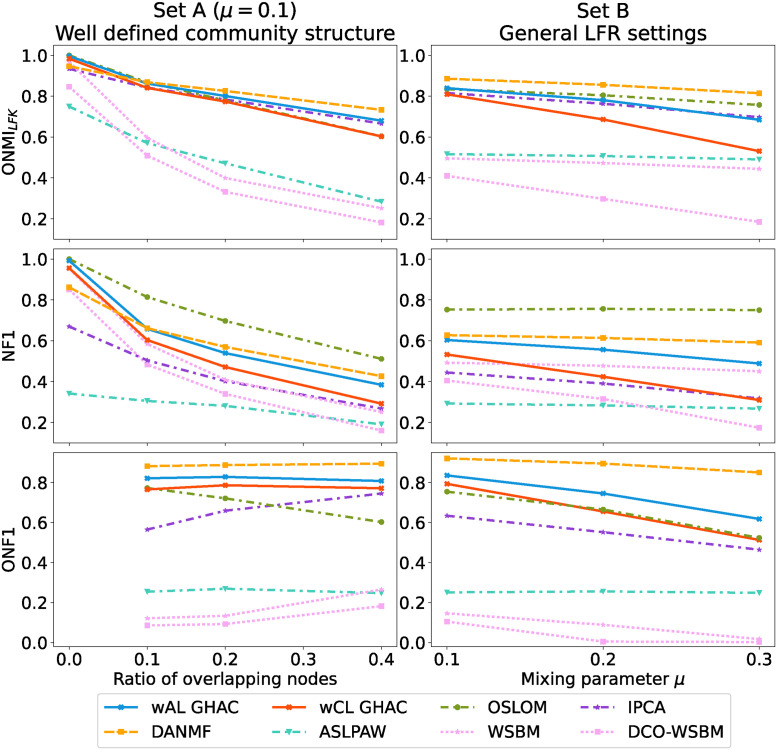
Performance evaluation of community detection methods on a set of LFR benchmarking networks.

Fig 6 portrays the performance of various community detection methods across two distinct LFR benchmark settings: set A with well-defined community structures with the mixing parameter *μ* = 0.1 and set B reflecting general LFR configuration with overlapping communities, described in detail in Section 5.1. An increase in the ratio of overlapping nodes leads to a decline in *ONMI*_*LFK*_ and NF1 scores for all methods.

DANMF tends to secure higher *ONMI*_*LFK*_ scores, with wAL GHAC and IPCA following closely. When evaluated using NF1, OSLOM appears to outperform other methods. For ONF1, which measures the correct detection of overlapping nodes, DANMF and wAL GHAC achieve the highest scores, indicating their effectiveness in this aspect. It should be emphasized that the optimal DANMF score resulted from a time-consuming optimization process, where the number of communities was finely tuned to maximize external community quality. This approach does not reflect typical real-world scenarios.

A direct comparison between the proposed GHAC methods reveals that wAL GHAC consistently surpasses wCL GHAC in all tested scenarios. This could be attributed to the difference in similarity definitions, with the weighted average linkage offering a more nuanced approach to community merging.

WSBM methods show diminished performance in high-overlap scenarios, a limitation partially highlighted in Peixoto’s work [28], possibly due to SBM’s core design not fully accommodating the complexities of overlapping communities.

### 5.5 Case study: OECD countries trade network

Studies of global trade networks reveal the complex interactions between economic activities and environmental impacts [54], outline preferential trade patterns [55], and reveal the hierarchical organization of product flows [56]. In this study, we examine the trade connections among current OECD member states and trading partners, utilizing the Balanced Trade Value for Total Product as an indicator of bilateral trade relations. The chosen dataset, Balanced International Merchandise Trade dataset [57], encompasses recorded trade values in US dollars from 2007 to 2018. The average annual trade value throughout this interval was the foundational metric to construct the network. To compensate for the skew resulting from vastly differing absolute trade values across nations, we normalized these figures against each country’s total trade volume. A threshold was established wherein only trade links accounting for at least 5% of a nation’s total international trade were considered significant enough to be included in the network. This study also addresses the inherent asymmetry in international trade relationships by converting directed trade links into undirected ones, utilizing the mean value of the two directional links to represent the strength of the bilateral trade relationship. This methodological approach ensures a more balanced representation of trade interactions within the network model. The constructed weighted network comprises 43 nodes, indicative of involved countries, and 187 edges, representing the significant trade relationships between these nations.

**Fig 7:**
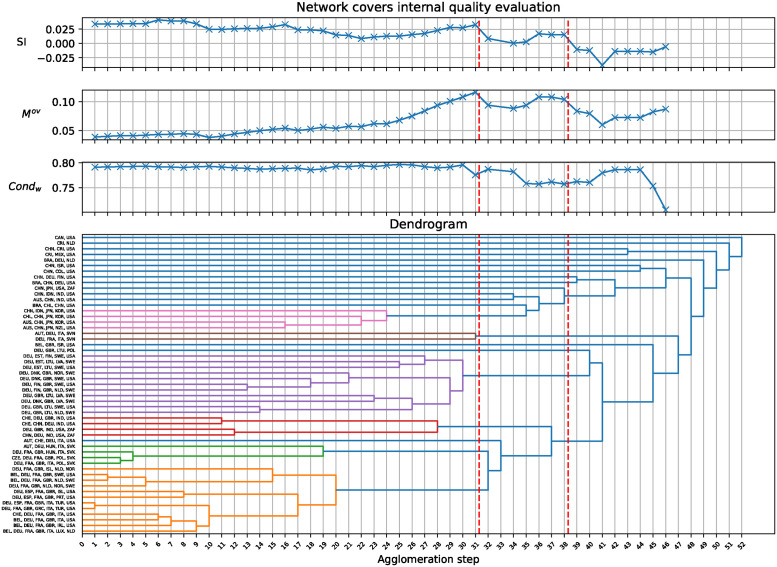
Analysis of OECD trade network via wCL GHAC. The highlighted peaks in internal quality are further illustrated as network covers in Fig 8.

**Fig 8:**
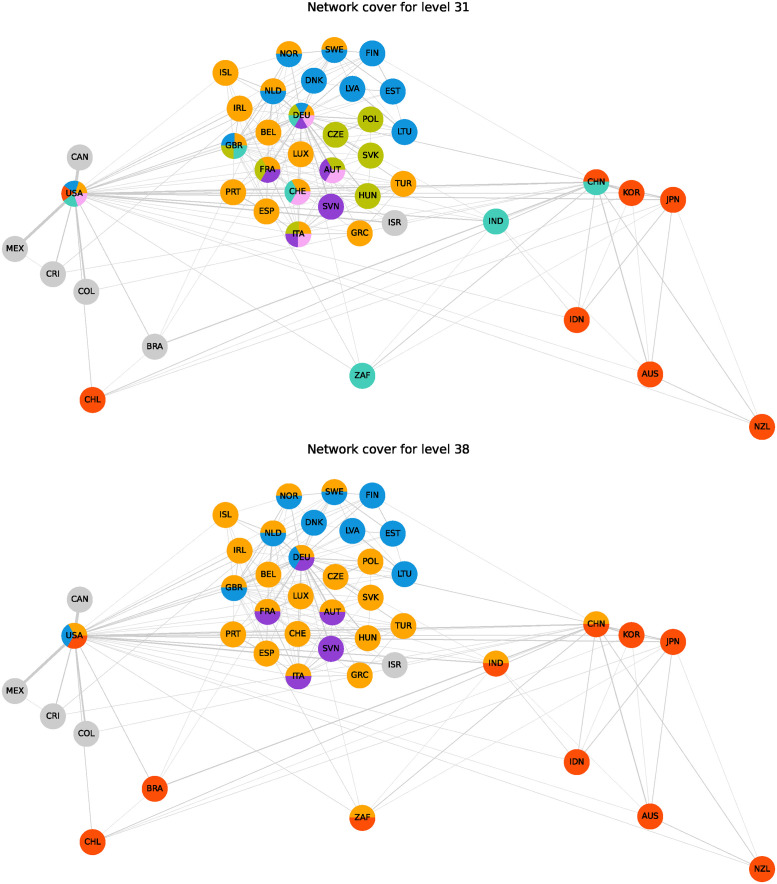
Network visualization of OECD member and partner trade relations using the wCL GHAC community detection method. It shows a nested property of green community (V4 Group and Core European economies) that is part of a broader transatlantic community (orange community in level 38). The layout approximates the geographic positioning of nations, employing a Noverlap strategy to enhance clarity.

Fig 7 shows the wCL GHAC method’s application to the OECD trade network. The internal community quality is monitored by the Silhouette index (SI), modularity (*M*^*ov*^), and conductance (*Cond*_*w*_) across the agglomeration steps in a dendrogram. Peaks in internal quality, marked by dashed lines, correspond to potential cuts for optimal community structure and are visually detailed in network covers illustrated in Fig 8. Additionally, the same figure highlights the nested structure of communities, where two local optima suggest different levels of granularity in the community structure.

The network visualization in Fig 8 shows the interconnectedness of trade relations among OECD countries and partners. The communities observed in level 31 can be associated with regions or shared economic interests, as follows:

- Western Europe and USA (orange): This community integrates most of Western Europe with the USA, indicating strong transatlantic trade ties and shared economic interests.
- Asia-Pacific and USA (red): Focuses on key Asia-Pacific economies alongside the USA, reflecting strong regional economic cooperation.
- Central European group (purple): Group of Central European countries, demonstrating tight regional integration and economic interdependence.
- Northern and Baltic Europe and USA (blue): Combining Northern and Baltic European countries with the USA and UK, this community underscores the significance of the Baltic Sea region for trade.
- Global Economic Powers (aquamarine): Includes major global economies such as the USA, UK, Germany, China, and India, highlighting a group of countries critical to global supply chains.
- Central Europe and USA (pink): Represents a smaller subset of Central European countries with the USA, indicating specialized economic partnerships.
- Visegrad Group (V4) and Core European economies (green): It reflects the V4 countries’ regional cooperation alongside key Western European powers, underscoring their central roles in Europe’s economic landscape.
- Brazil, Canada, Colombia, Costa Rica, Israel, Mexico (gray): Each country stands alone as a single-community member, suggesting unique trade profiles or significant bilateral relationships not covered by the broader communities.

Between levels 31 and 38 in Fig 8, the dynamics of the community structure reveal a clear nesting pattern where smaller, more granular communities merge into larger, more comprehensive groups. Specifically, the Visegrad Group (V4) and Core European economies (green) community, initially distinct at level 31, merges into a broader transatlantic community, Western Europe and USA (orange), by level 38. This transition reflects the deepening trade ties between these regions and their integration into a larger transatlantic economic framework. This process shows how finer regional communities merge into larger structures, highlighting the multi-level nature of trade relationships within the network. Meanwhile, other communities, such as Northern and Baltic Europe and the USA (blue), maintain their stability, demonstrating the persistence of some regional ties even as other communities consolidate. Additionally, the Asia-Pacific and Americas (red) community extends to include Brazil, highlighting Brazil’s increasing integration into these global economic regions.

## 6 Conclusion

A hierarchical method for overlapping community detection in weighted networks has been introduced. Optimizing the methodology for cut selection of the dendrogram was the key part of this study. An extensive examination of internal quality metrics for community evaluation has identified the Silhouette index as the best criterion, as evidenced by its high correlation with external evaluations in the LFR benchmark. Comparative analyses reveal that wAL GHAC and wCL GHAC outperform various established community detection methods, especially in configurations involving overlapping communities. The wAL GHAC method, in particular, exhibits enhanced performance compared to wCL GHAC due to its comprehensive consideration of node pair distances within community structures. The OECD data demonstrate the usefulness of a hierarchical insight into overlapping community structure for the trade network.

Future research will aim to explore the quality of hierarchical structures more rigorously, focusing on quantitative evaluation. In applying our method to the OECD networks, which are naturally directed, we encountered methodological constraints. This required us to transform these networks into an undirected format. Subsequent research will focus on modifying the proposed method to process directed networks effectively. We suppose that *CT*−distance and the proposed dissimilarities for the GHAC method are already applicable to directed networks. Still, the understanding of communities in directed networks is a complex issue that needs further detailed study.
